# Differential Effects of Alcohol Policies Across Race/Ethnicity and Socioeconomic Status

**DOI:** 10.35946/arcr.v45.1.02

**Published:** 2025-01-14

**Authors:** Nina Moreno, Roland S. Moore

**Affiliations:** 1 Seed Collaborative, Inglewood, California; 2 Prevention Research Center, Pacific Institute for Research and Evaluation, Berkeley, California

**Keywords:** alcohol, alcohol control policy, alcohol-related disorders, health equity, health inequities, socioeconomic factors, ethnicity, health impact assessment

## Abstract

**PURPOSE:**

Sociocultural characteristics, including race/ethnicity and socioeconomic status (SES), may affect individuals’ attitudes and norms regarding alcohol use and treatment as well as their access to emerging health knowledge, innovative technologies, and general resources for improving health. As a result of these differences, as well as social determinants of health such as stigma and uneven enforcement, alcohol policies may not benefit all population subgroups equally. This review addresses research conducted within the last decade that examined differential effects of alcohol policies on alcohol consumption, alcohol harm, and alcohol treatment admissions across racial/ethnic and socioeconomic groups.

**SEARCH METHODS:**

The authors used the following Boolean phrase search terms to assess the association between race/ethnicity and outcomes: (“alcohol policy” OR “alcohol policies”) AND (“race” OR “ethnicity” OR “first nations” OR “African American” OR “Hispanic American” OR “Latino American” OR “Asian American” OR “Native American”). Association with SES was assessed using these terms: (“alcohol policy” OR “alcohol policies”) AND (“socioeconomic” OR “class”) AND (“effect” OR “impact” OR “outcome”). Both searches were conducted on August 28, 2023, using advanced search in seven EBSCOhost research databases: (1) EBSCO Biomedical Reference Collection: Corporate; (2) EBSCOhost E-Journals; (3) EBSCO MEDLINE Complete; (4) SocINDEX with Full Text; (5) APA PsycInfo; (6) LISTA (Library Information Sciences and Technology Abstracts); and (7) GreenFILE. Inclusion criteria for both searches were: (1) publication dates between 2014 and 2023; (2) peer-reviewed research articles; (3) data disaggregated by racial/ethnic and/or SES subgroups; and (4) English language only.

**SEARCH RESULTS:**

The racial/ethnic search produced 64 articles, of which 14 were reviewed as relevant to this study and 50 were excluded. The SES search generated 100 articles, of which 18 were reviewed as relevant to this study and 82 were excluded. Eight of the studies identified by these two searches overlapped (i.e., included both racial/ethnic and SES outcomes), resulting in a total of 24 articles included in this review.

**DISCUSSION AND CONCLUSIONS:**

Relying upon data from both U.S. and international research, the identified studies focused on differential effects of financially focused alcohol control policies (e.g., taxation and minimum unit pricing policies) as well as access/availability reduction policies (e.g., those governing outlet density, on-/off-premise sales, and establishment licensing). Several studies concluded that price increases via taxes or minimum unit pricing might be particularly effective in reducing the risk of alcohol-related harms in low-income/low-SES populations. Limitations of the present review include lack of standardization in the ways that SES was measured and the difficulty of measuring policy enforceability. Studies focused on differential effects of alcohol control policies across racial/ethnic groups demonstrated complex associations and the need to conduct further research that identifies better ways to reduce policy-induced health disparities across diverse populations.

Altering the alcohol policy environment to maximize health and well-being for the largest number of people is a demonstrably effective public health principle.^1^ Alcohol control legislation and other policies—constituting primary prevention—aim to boost population health comprehensively^2^ via interventions such as alcohol taxes, laws prohibiting driving under the influence, and minimum age of sales restrictions for alcohol.^3^,^4^ However, despite the implicitly egalitarian aims of population-level public health policies, a large body of evidence shows that laws created for all do not affect every population subgroup to the same extent.^3^,^4^ Intervening external factors, such as differential enforcement of these laws, as well as structural barriers to implementation may interact with cultural perspectives and intrapersonal factors, including higher stress levels, to produce different levels of engagement with and benefit from these laws.^5^,^6^

This review evaluates research published in peer-reviewed journals over the past decade regarding the differing effects of alcohol control policy interventions on two population subgroups often bearing a disproportionately heavy burden of alcohol-related problems: those with low socioeconomic status (SES), and racial and ethnic population subgroups. Although higher SES and non-Hispanic White race/ethnicity are associated with higher overall drinking levels, the scientific literature demonstrates that alcohol-related harms disproportionately affect members of disadvantaged groups, including members of other racial/ethnic groups and individuals with lower SES. The theoretical mechanisms underlying these seemingly contradictory findings include factors such as consumption of larger amounts per drinking occasion; fewer protective resources (e.g., housing assistance, food security); and structural stigma, including uneven enforcement of alcohol control policies.^7^

Because alcohol consumption can result in serious harms to individuals, families, schools, workplaces, and communities, a wide variety of alcohol control policies have been implemented over time to reduce consumption and, in turn, alcohol-related harm.^1^,^3^ Examples of financially focused alcohol control policies include excise taxes (i.e., taxes levied on the sale of specific goods/services, such as alcohol); minimum unit pricing (MUP), which specifies legal floor prices for a unit of alcohol below which it cannot be sold; and price promotion bans.^1^,^3^ Such financial alcohol control policies can profoundly improve public health in general, even as they fall short of affecting subpopulations equitably. A 2023 systematic literature review and meta-analysis by Kilian et al. of studies published between 2000 and 2022 examined the ways in which new or changed financial alcohol control policies may reduce alcohol use at both general population and population subgroup levels.^8^ The authors identified 36 eligible studies and concluded that doubling alcohol taxes or initiating MUP reduced alcohol consumption by 10% in the general population. However, changes in consumption were greater for people with lower incomes who used alcohol, although the results were inconclusive for racial/ethnic groups.^8^ The quantitative rigor of this meta-analysis points to the power of these financial alcohol control policies to induce widespread reductions in alcohol consumption and related harm.

Another review article, published 8 years earlier, summarized extensive research on financially focused alcohol policy effects conducted in Finland and established an evidence base for examining differential effects of financial policy changes on disadvantaged population groups.^9^ Mäkelä et al. reviewed Finnish studies on the connections between SES, alcohol use, related harm, and possibilities for intervention by means of alcohol pricing. The authors concluded that the socioeconomic differences in severe alcohol-related harm were great, and that these differences had widened in the 2 decades preceding their analysis. They also noted that alcohol-related mortality had strongly contributed to both the level and widening of socioeconomic differences in life expectancy. Furthermore, both in 2004—when Finnish alcohol prices were abruptly reduced as the country joined the European Union—and in the longer term with more gradual changes in lowest prices of alcohol, the groups with the lowest SES were most affected, particularly men. However, these effects were sometimes weak and not fully consistent by gender and across different measures of harm.^9^

Although these two reviews make a strong case for focusing on increasing MUP and taxation as the most powerful policy levers to reduce alcohol consumption and related harm, other policy tools, at least theoretically, also hold the potential to reduce alcohol-related problems. For example, the place or time of alcohol sales can be limited by imposing density or location restrictions (e.g., zoning laws prohibiting new alcohol licenses in an area with numerous existing outlets), temporal limits such as restricted hours of sales, and age limits (e.g., the nationwide minimum legal drinking age of 21 in the United States). The underlying mechanism across these policies is the reduction of alcohol availability, which tends to reduce individual and collective consumption and subsequent harm from drinking.^10^,^11^

Other alcohol control policies—inspired by epidemiological evidence and laboratory studies demonstrating diminished response time during intoxication—focus on workplace and transportation safety. They include zero-tolerance policies in workplaces; impaired driving policies, such as those analyzed and promoted by the National Highway Traffic Safety Administration (https://www.nhtsa.gov/laws-regulations/guidance-documents#52891); checks of blood alcohol concentration limits at random enforcement checkpoints; and legislative advocacy efforts by groups such as Mothers Against Drunk Driving.^12^ Additional alcohol control policies aim to change norms and increase knowledge via advertising restrictions and warning labels. Measures that have been used to determine the outcomes of these policies (with numerous caveats about their accuracy) include, but are not limited to, statistics on alcohol-related trauma and liver disease from hospital discharge databases, sales rates to underage or apparent underage decoys attempting alcohol purchases, and large-scale epidemiological self-report survey findings on health-related data.

The stated goal of these kinds of policies is population-level primary prevention; however, a growing body of evidence is showing that different subpopulations react to these policies differently. Thus, this article reviews some financially based alcohol control policies as well as access/availability reduction policies that, following the reviews by Kilian et al.^8^ and Mäkelä et al.,^9^ show promise in reducing alcohol-related harm among populations with low SES. It also identifies some gaps in the literature regarding effective alcohol control policies that show clear evidence of improving health of disadvantaged racial/ethnic groups, and how those gaps might be addressed through additional refined research.

## Search Methods Employed

Two searches were conducted on August 28, 2023, for English-language, peer-reviewed articles published between 2014 and 2023 that examined differential outcomes of alcohol control policies in populations with different race/ethnicity or SES. The race/ethnicity search used the following Boolean/phrase search terms: (“alcohol policy” OR “alcohol policies”) AND (“race” OR “ethnicity” OR “first nations” OR “African American” OR “Hispanic American” OR “Latino American” OR “Asian American” OR “Native American”). The SES search used the following terms: (“alcohol policy” OR “alcohol policies”) AND (“socioeconomic” OR “class”) AND (“effect” OR “impact” OR “outcome”). To strive for relatively complete coverage of the literature, these search terms were used in an advanced search in seven EBSCOhost Research databases: (1) EBSCO Biomedical Reference Collection: Corporate; (2) EBSCOhost E-Journals; (3) EBSCO MEDLINE Complete; (4) SocINDEX with Full Text; (5) APA PsycInfo; (6) LISTA (Library Information Sciences and Technology Abstracts); and (7) GreenFILE. Nevertheless, these searches cannot be considered comprehensive but are instead suggestive, because additional search terms would have captured additional significant articles.

The inclusion criteria for this review were: (1) publication dates between 2014 and 2023; (2) peer-reviewed research study; (3) data disaggregated by racial/ethnic and/or SES subgroups; and (4) English language only. Accordingly, exclusion criteria were: (1) publication date prior to 2014; (2) non-peer reviewed articles; (3) aggregated population data only; and (4) languages other than English.

## Results

### Results of the Literature Search

The racial/ethnic search produced 64 articles, of which 14 were reviewed as relevant to this study and 50 were excluded because they did not meet the inclusion criteria. The SES search generated 100 articles, of which 18 were reviewed as relevant to this study and 82 were excluded because they did not meet the inclusion criteria. Eight articles were identified by both searches because they included both racial/ethnic and SES outcomes; therefore, a total of 24 articles were included in the review. Appendix 1 lists the selected articles and their main study characteristics, grouped by focus on SES, racial/ethnic groups, and both SES and racial/ethnic groups.

The included studies reported data from the United States (11 articles),^6^,^13^–^22^ Australia (seven articles),^23^–^29^ India (one study),^30^ Canada (one study),^31^ Malaysia (one study),^32^ and the United Kingdom (one study).^33^ Moreover, three papers reported data from multiple international sources.^34^–^36^ Ten papers focused on the influence of SES,^13^,^23^,^26^,^27^,^29^,^31^,^33^–^36^ six studies focused on race/ethnicity,^15^,^17^,^18^,^20^,^21^,^24^ and eight studies assessed the impact of both SES and race/ethnicity.^6^,^14^,^16^,^19^,^22^,^25^,^30^,^32^

The identified studies examined differential effects of economic alcohol control policies, including financially focused, access/availability reduction, and transportation safety policies. Rather than encompassing all alcohol policies, most articles tended to focus on specific policies and their differential effects on subpopulations. For example, six articles addressed financially focused alcohol policies;^16^,^27^–^29^,^32^,^35^ four studies addressed alcohol access/availability reduction policies;^22^,^23^,^31^,^34^ five studies explored national alcohol control policies, national government regulations, and strategies implemented to manage, regulate, and control various aspects of national interest, including, but not limited to, public health, environmental protection, economic stability, and social welfare;^8^,^9^,^21^,^25^,^32^ and one study concerned alcohol licensing policy.^23^

### Results of the Reviewed Studies

#### Differential effects of financially focused policies

As previously mentioned, Kilian et al. concluded that by doubling alcohol taxes or initiating MUP, alcohol consumption was reduced by 10% in the general population.^8^ Economic modeling has shed light on alcohol pricing effects on different SES groups. For example, Jiang et al. used estimated price elasticities (i.e., how sensitive the demand for alcohol is to changes in its price) to model the effects of proposed pricing policies on consumption for 11 beverage categories among subpopulation groups in Australia.^27^

Mean and percentage changes in alcohol consumption were estimated for each scenario across subgroups based on drinking level (moderate, hazardous, and harmful), age (younger, middle, and older), and income (lower, middle, and higher). The policy scenarios evaluated included: (1) increasing the excise rate by 10% for all off-premise beverages; (2) replacing the wine equalization tax (a tax levied on the wholesale value of wine) with a volumetric excise rate (a type of excise tax calculated based on the volume of wine rather than its value) equal to the current spirits tax rate; (3) applying a uniform excise tax rate to all beverages equal to, or 10% or 20% higher than, the current spirits tax rate; and (4) introducing an MUP on all beverage categories at $1.00, $1.30, or $1.50 per 10 grams of alcohol (an Australian standard drink). The analysis demonstrated that the lower-income group consumed a higher proportion of alcohol for under $1.30 per 10 grams than did higher-income groups.^27^ Similarly, Holmes et al. found evidence through modeling research that establishment of MUP for alcohol in the United Kingdom would reduce harmful drinking (i.e., > 50 units per week for men and > 35 units per week for women) for individuals with low incomes, whereas people who drank moderately (i.e., ≤ 21 units per week for men and ≤ 14 units per week for women) were not particularly affected by the policy change.^33^ This observation suggests that MUP has the potential to improve in particular the health of people with low SES who drink at harmful levels.^33^

Patterns similar to those identified in modeling studies also were found in large-scale survey analyses. Casswell et al. used a cross-sectional, general population survey that included measures of consumption variables (e.g., quantity and frequency of consumption on a typical occasion), policy variables (e.g., purchasing behavior, time of purchase, prices, liking of alcohol ads), and demographics.^35^ The analysis concluded that individuals reporting having fewer years of education purchased lower-priced alcohol than did their more educated peers, which mediated drinking more frequently in off-premise establishments among the less-educated group.^35^

Jiang et al. used multivariable logistic regression of data on older adults from the Australian 2016 National Drug Strategy Household Survey to assess the correlates of risky drinking,^29^ defined as a pattern of alcohol consumption that increases the likelihood of adverse health outcomes and other harmful consequences for the person who drinks or others. The study found that 22% of low-income participants (versus 31% of highest-income participants) drank at risky levels. Nonetheless, Jiang et al. argued for the employment of interventions aimed at older adults, both low- and high-income alike, to move toward reducing alcohol consumption via pricing policies.^29^

One other relevant study addressed the relationship between MUP/pricing policies, drinking levels, and SES, establishing that low-SES groups responded to increases and decreases in alcohol pricing. Jiang and colleagues estimated own- and cross-price elasticities of alcohol demand (i.e., how much of an item people are willing to buy when the price changes of that item [own-price] or of competing products [cross-price]) among 11 subcategories of beverages based on beverage type and on- or off-premise supply, using cross-sectional data from the Australian arm of the International Alcohol Control Survey 2013.^26^ Further elasticity estimates were derived for subgroups of people who consumed alcohol based on their drinking and income levels. Considering both income levels and the degree to which respondents engaged in risky drinking, the analyses indicated that people with higher levels of risky drinking or lower income appeared to be more responsive to price than were people who drank moderately or had higher income.^26^

Two studies established that an increase in alcohol taxes was associated with a decrease in consumption and alcohol-related harms among low-SES and minoritized racial/ethnic groups.^16^,^21^ Naimi et al. modeled the increased net cost of alcohol (i.e., product plus tax) from a series of hypothetical state alcohol tax increases for all 50 U.S. states using data from the 2011 Behavioral Risk Factor Surveillance System, IMPACT Databank, and the Alcohol Policy Information System.^16^ Costs were assessed by drinking pattern (e.g., excessive versus non-excessive) and by sociodemographic characteristics. Excessive drinking was defined as binge drinking (consuming five or more drinks for men or four or more drinks for women on one occasion), heavy drinking (consuming an average of more than two drinks per day for men or more than one drink per day for women), or any alcohol consumption by respondents ages 18 to 20 (i.e., adults under the minimum legal drinking age). Non-excessive drinking was defined as consuming alcohol in the past 30 days but not classifying as excessive drinkers. The analyses demonstrated that members of low-income households and racial/ethnic minorities, including those who did not drink excessively, purchased less alcohol (i.e., paid less in per capita and aggregate costs) following tax increases, compared with non-Hispanic White people or those with higher incomes. These observations suggest that increasing the price of alcohol might be a particularly effective way to reduce the risk of alcohol-related harms in low-income populations.^16^

Subbaraman and colleagues examined 15 years’ worth of state-level taxation of different categories of alcohol and identified some ethnicity-specific variations on the general finding that increased taxes were associated with a decrease in alcohol consumption.^21^ Higher taxes on beer led to lower alcohol volume consumed and fewer alcohol-related consequences for Black women in particular, whereas higher taxes on spirits reduced alcohol volume consumed among Hispanic women and men.^6^

Conversely, another study established that low alcohol taxes were associated with an increase in alcohol consumption among low-SES groups, providing further evidence that taxes can serve as a tool to prevent problems with alcohol. In a study conducted in Malaysia, Shoesmith et al. showed that inexpensive, untaxed alcohol (called Montoku) was consumed by people with very heavy episodic drinking living in a region with high economic deprivation.^32^ The authors argued that levying an excise tax in the form of a specific rate per liter of alcohol was more effective than an ad valorem excise tax (which taxes alcohol according to value) at reducing alcohol consumption, particularly among people from lower SES groups.^32^

#### Differential effects of access/availability reduction policies

The second main policy approach focuses on regulating alcohol access and availability, including location-based policies (e.g., zoning to address alcohol outlet placement) and temporal-based policies. Three studies identified in the literature search established associations between alcohol access or availability reduction policies—namely, outlet density, operation hours, and on-/off-premise policies—and social and health disparities among SES and racial/ethnic groups. Badland et al. found that lower outlet density was associated with better self-reported health,^23^ whereas Myran et al. determined that an increase in hours of operation and an increase in the number of alcohol outlets were associated with an increase in alcohol-related visits to the emergency department (ED).^31^ Additionally, Trangenstein et al. established that census block group (CBG) neighborhoods with a history of disinvestment or redlining (e.g., denial of mortgages or sales to Black, Indigenous, and other racial/ethnic minorities) were associated with a higher concentration of businesses/establishments that sell alcoholic beverages for consumption off the premises where they are sold (i.e., increased off-premise clusters).^22^ These studies were consistent with earlier research on the association of off-premise outlet density and health harms resulting from excessive drinking.^37^

Trangenstein et al. elaborated that alcohol outlets often cluster in predominantly non-White neighborhoods, resulting in increased alcohol availability for non-White racial/ethnic groups;^22^ this practice often is labeled as structural racism.^38^,^39^ The study used 2016 liquor license data (*n* = 1,204) from Baltimore City, Maryland, and demographic data from the American Community Survey for a multiple logistic regression analysis to compare CBGs (*n* = 537) inside and outside of four types of outlet clusters: total, on-premise, off-premise, and combined on-/off-premise outlets. The analyses found that the most robust predictor of alcohol outlet cluster membership was a history of redlining. CBGs that were redlined had 7.3 times the odds of being in an off-premise cluster, 8.1 times the odds of being in an on-premise cluster, and 8.6 times the odds of being in a combined on-/off-premise cluster compared with CBGs that did not experience redlining. In addition, level of economic investment (marked by vacant properties) appeared to be a key characteristic affecting CBG cluster membership. Thus, CBGs with racial/ethnic or socioeconomic advantage had higher odds of being in on-premise clusters, whereas CBGs marked by disinvestment had higher odds of being in off-premise clusters.^22^

To determine how alcohol availability affected alcohol-related harm, Myran et al. investigated the association between alcohol availability and alcohol-attributable ED visits in the province of Ontario, Canada, before and after deregulation of the number of alcohol outlets.^31^ In December 2015, Ontario allowed large privately owned grocery stores to apply for a license to sell wine, beer, and cider in specified aisles or sections within stores; this resulted in 450 licenses by 2020, increasing the number of off-premise outlets in the province by 29%. The study found that alcohol-attributed ED visits increased by 18% over the study period, which was more than twice the increase for all ED visits. In particular, increased hours of operation and numbers of alcohol outlets within defined geographic regions (forward sortation areas [FSAs]) were positively associated with higher rates of alcohol-attributable ED visits. The increase in alcohol-attributable ED visits was 6% greater in FSAs that introduced alcohol sales in grocery stores following deregulation compared with FSAs that did not (incident rate ratio = 1.06; 95% confidence interval [1.04, 1.08]). Marginalization—the process by which certain groups or individuals are pushed to the edge of society, limiting their access to resources, opportunities, and rights— appeared to be independently associated with alcohol-attributable ED visits. Thus, each quintile increase in the residential instability and material deprivation of an FSA was associated with a 25% and 18% higher rate of ED visits, respectively.^31^

Badland et al. addressed the association between alcohol outlets as well as on-/off-premise policies and SES on the one hand and self-rated health on the other hand.^23^ The authors generated spatial measures of alcohol outlet density in a geographical information system and linked them with health survey data drawn from 3,141 adults living in metropolitan Melbourne, Australia. Logistic regression analysis was used to examine associations between 12 spatial measures of alcohol outlet density, self-rated health, and area-level disadvantage. Alcohol outlet density and self-rated health associations varied by area-level disadvantage. For people living in more disadvantaged areas, not having retail establishments authorized to sell alcoholic beverages for off-premise consumption available within 800 meters of their residence, or not having retail establishments authorized to sell alcoholic beverages for on-premise consumption available within 400 meters of their residence, were protective of self-rated health.^23^

Additionally, Casswell and colleagues analyzed a cross-sectional general population survey to understand direct and mediating pathways to alcohol consumption.^35^ Study participants comprised 1,900 people ages 18 to 65 who lived in households with landline phones and who had consumed alcohol in the past 6 months. The survey assessed typical alcohol quantities consumed per occasion, frequency of both off- and on-premise drinking, prices paid for alcohol, time of purchase, gender, age group, years of education, personal income, and liking of alcohol advertisements. The analyses found that participants with fewer years of education paid lower prices for alcohol, and this mediated more frequent off-premise drinking among this group.

#### Differential effects of national and other alcohol control policies

Five studies argued for national alcohol control policies in the mitigation of alcohol consumption, alcohol intoxication, assault/vandalism, and driving-related harm caused by another person who had been drinking.^13^,^14^,^25^,^30^,^36^ Balasubramani et al. conducted a secondary analysis of data from India’s National Family Health Survey-4 with a large sample size of 103,411 men and 699,686 women.^30^ Findings indicated that most of the districts with high alcohol consumption, especially by women in alcohol hot spots (i.e., districts with high alcohol prevalence surrounded by districts with high alcohol prevalence), also had a high proportion of scheduled Tribe members in the population—Indigenous community members who are recognized by the Constitution of India and are among the most socioeconomically disadvantaged individuals in the country. The multilogistic regression analysis also indicated a strong association between proportion of scheduled Tribe members in a population and alcohol consumption by men (adjusted odds ratio: 1.4) and women (adjusted odds ratio: 5.8); moreover, 75% of women who consumed alcohol were from scheduled Tribes. Among women, high alcohol consumption also was seen among the poorest people, individuals with no education, and manual/agricultural workers. The authors argued that the lack of a national policy on alcohol control, interacting with racial/ethnic differences, was the major reason for the high alcohol consumption across India.^30^

Leal-Lopez et al. studied lifetime alcohol consumption, weekly alcohol consumption, and lifetime drunkenness among 671,084 adolescents from 33 European and North American countries/regions over a 4-year period, taking into account the cumulative strengths of each country’s alcohol access restriction policies.^36^ Findings indicated that socioeconomic inequality (i.e., adolescents who perceived their families to be poor were more likely to get drunk) persisted and even increased across survey years. Leal-Lopez et al. argued that a combined alcohol control policy or an integrated approach that utilizes multiple strategies and measures to reduce alcohol-related harm could help in tackling this socioeconomic inequality.^36^

Greenfield et al. assessed the associations between U.S. state alcohol policies and alcohol-related harms to others.^14^ The analyses found that in states with more effective laws for reducing binge drinking and impaired driving (i.e., as indicated by higher scores on the Alcohol Policy Scale), people with a college degree or higher, particularly those under age 40, were less likely to experience alcohol-related harms compared to others.

With reference to state policies designed to reduce the harms caused by heavy drinking, Cook et al. found that lower state alcohol policy strength scores were not directly related to alcohol-related harm, but did have significant indirect effects on increased risks for assault/vandalism and driving-related harm through increased binge drinking prevalence.^13^ The authors suggested that a more stringent alcohol policy environment might reduce assault/vandalism and driving-related harm due to other people who had been drinking by lowering state binge drinking rates. As a cautionary observation, Conigrave et al. found that drinking patterns in Indigenous Australian populations varied within and between communities and that policies to reduce high-risk drinking need to account for this variability.^25^

The authors argued that attention needs to be paid to nuanced cultural and social contexts, rather than implementing a one-size-fits-all policy for distinctive communities.^25^

Another realm of research developing in the last decade concerns clusters of policies designed to reduce drinking during pregnancy, which have had mixed results in different populations. Two studies by Roberts et al.^17^,^18^ found that for pregnant Black women, priority treatment (i.e., focused support/services for pregnant women and women with children who have faced disadvantages, discrimination, and/or barriers to access) was associated with less heavy drinking, whereas for pregnant White women, prohibitions on criminal prosecution of pregnant women who drank were associated with less heavy drinking.^18^ However, state policies regarding alcohol use during pregnancy had unintended adverse effects for White women by increasing rates of preterm births or low birth weight, whereas those same policies were more beneficial for reducing preterm births in Black women.^17^

The mixed results reported in this section indicate promise in reducing alcohol-related harm among low-SES subsets of populations that have emerged in recent scholarship. However, some gaps remain in the literature regarding effective alcohol control policies that have yet to show clear evidence of improving health of disadvantaged racial/ethnic groups; additional refined research may address this circumstance.

## Conclusions

Echoing the review of Kilian et al.,^8^ the present review focuses on financially based alcohol control policies, which compared with other types of alcohol control policies hold the greatest promise for substantially reducing problems related to alcohol, including among lower SES populations. The studies reviewed here relied on several methodological approaches, including combining large survey datasets with contextual information from various sources (e.g., the National Institute on Alcohol Abuse and Alcoholism’s Alcohol Policy Information System^40^) about changing alcohol control policies at the state or national level, as well as simulation approaches such as agent-based modeling. One of the recurring conclusions was that when taxes or MUP were applied to alcohol prices, the lower-SES groups were most likely to gain health benefits, even as many other groups gained health benefits as well. Furthermore, comparing the relative strength of alcohol policies from state to state, the policy suites held by states that were assessed to be the strongest alcohol policies also featured less inequality for lower SES groups.

### Review Limitations

One of the many limitations of this review is the lack of standardization among studies in measures to operationalize the different effects of various policies, making precise comparisons difficult. However, as Kilian et al. indicated, there are now enough studies in some specialized areas of economic research to suggest how alcohol MUP, taxation, and restricted hours of sale affect different SES and racial/ethnic groups in distinct ways.^8^ Another persistent challenge is that when comparing policies across states, or within smaller jurisdictions, enforceability or actual enforcement may be more difficult to measure.

With respect to scholarly languages, the inclusion criterion of English-only research may have inadvertently excluded some Scandinavian-language articles, such as those by Bruun et al.^2^ and others, that established the analytical framework for assessing alcohol control policies at the population and subpopulation levels. Nevertheless, the majority of Scandinavian research articles in recent years have been published in English.

Limitations also exist in the way SES and racial/ethnic membership are determined. Thus, SES is not measured solely by income; educational levels also contribute to the concept. However, the majority of economic papers discussed focused more on income than other overlapping variables, including educational levels.

In terms of racial/ethnic differences, most of the papers cited that considered race and ethnicity tended to focus on non-Hispanic Whites, Blacks, and Latinx populations and excluded other groups, such as Asian Americans, Native Hawaiians, and American Indians and Alaska Natives. It is worth noting that one study addressed the impact of national alcohol control policies on race/ethnicity. Clough et al. studied the impacts of alcohol management plans in Indigenous communities in Queensland, Australia.^24^ This study found that participants attributed reduced violence and improved community amenity/quality of life to such plans, particularly for “very remote” Indigenous communities.^24^

Finally, the particular search terms used for this review are certain to have missed important scholarly contributions. However, it is likely that the studies summarized in Appendix 1 represent at least a suggestive array of the wider range of research on this topic that continues to develop.

### Areas for Future Research

Although this review focused on the ways in which alcohol policies differentially affect diverse racial/ethnic and SES groups, other significant and related areas may be worth investigating in the future. For example, in recent years, the National Institutes of Health has expanded the definition of minority groups to include sexual and gender minorities. More recently, people with disabilities also have been considered a federally recognized population with health disparities, opening the door to research funding announcements with a particular focus on health policies and their differential impact on, among others, people with disabilities. It is also worth considering the differential effects of policies on individuals of different ages, as availability restrictions on alcohol purchases are particularly salient for people under age 21; however, neurological and physiological changes throughout one’s lifetime make alcohol consumption riskier at either end of the lifespan or during pregnancy.

Even as the research on alcohol control policies develops further, many facets of the relationship between the policies and outcomes of diverse racial/ethnic and socioeconomic groups remain unexplained. Thus, mixed-method research on the underlying reasons for inequality in the effects of specific policies would help to create a greater understanding of the mechanisms driving these differences. A persistent concern is that although non-Hispanic Whites have among the highest rates of heavy and binge drinking, other population groups— including Blacks, American Indians, and Alaska Natives— experience higher rates of deaths from causes related to binge drinking.^41^ Further research can help disentangle structural causes as well as the impact of harmful alcohol use on allostatic load, which is associated with minoritized status, and how alcohol policies can address structural inequities and the impact of harmful alcohol use on Blacks, American Indians, and Alaska Natives.

**KEY TAKEAWAYS**
- Alcohol control policies that increase alcohol taxes or establish minimum unit pricing have been demonstrated to reduce alcohol consumption at the population level.
- Increased financial alcohol control policies also reduce alcohol consumption and harm among low-socioeconomic status (SES) subgroups; however, the evidence is inconclusive for racial/ethnic groups.
- This literature review demonstrates current limitations in comparing effective alcohol control polices and their enforcement across different-sized jurisdictions, as they may differentially affect subpopulations, including racial/ethnic and low-SES groups.
- Future research incorporating mixed-method analyses of structural racism and stress associated with minoritized status can help shed more light on the mechanisms underlying differential effects of alcohol policies on varied subpopulations.

## Figures and Tables

**Appendix 1:** Differential Effects of Alcohol Policies on Varied Socioeconomic Status (SES)-Based and Racial/Ethnic Populations

Study and Publication Year^*^	Setting (Place, Time)	Type of Study	Alcohol Policy Outcomes Assessed	Findings
**Studies including SES-based subgroups**				
Badland et al. 2016 23	Australia, 2014	Secondary data collection and analysis: Review of state-level spatial alcohol policies	Alcohol outlet density/clusters; on-/off-premise policies	**Lower outlet density was associated with improved health**. Alcohol outlet density and self-rated health associations varied by area-level disadvantage. For those living in more disadvantaged areas, not having off-premise outlets available within 800 meters of their residence, or on-premise outlets available within 400 meters of their residence, were protective of self-rated health.
Casswell et al. 2016 34	International, July and October 2011	Primary data collection and analysis: Cross-sectional general population survey	Alcohol outlet density/clusters, on-/off-premise policies; minimum unit price (MUP)/pricing policy	**Introducing/increasing MUP decreased alcohol consumption; decreasing MUP increased alcohol consumption**. Among people who consumed alcohol off-premise, price mediated both the association between education and frequency of drinking and the association between income and frequency of drinking.
Casswell et al. 2018 35	International, 2011–2016	Primary data collection and analysis: General population surveys	MUP/pricing policy	**Introducing/increasing MUP decreased alcohol consumption**. Individuals with lower education levels paid lower prices, and this mediated drinking more frequently off-premise among this group.
Cook et al. 2021 13	United States, 2000, 2005, 2010, and 2015	Primary data collection and analysis: Telephone interviews	National alcohol control policies	**National alcohol control policies mitigated harmful alcohol behaviors**. Low SES was a risk factor for family problems due to alcohol-related harms to others.
Holmes et al. 2014^33^	United Kingdom, 2001–2009	Secondary data collection and analysis	MUP/pricing policy	**Introducing/increasing MUP decreased alcohol consumption**. Raising MUPs for alcohol affected different SES groups differentially, with people with low incomes who drank heavily more likely to reduce drinking due to larger costs to them.
Jiang et al. 2016 26	Australia, 2013	Secondary data collection and analysis	MUP/pricing policy	**Increasing alcohol taxes or introducing an MUP can reduce alcohol demand**.
Jiang et al. 2020a 27	Australia, 2013	Secondary data collection and analysis	MUP/pricing policy	**Introducing/increasing MUP decreased alcohol consumption**. The lower-income group consumed a higher proportion of alcohol for under $1.30 per 10 grams of alcohol than did higher-income groups, although the result was statistically insignificant. Individuals with lower income who consumed alcohol at harmful levels drank more off-premise cask wine than did other income subgroups.
Jiang et al. 2020b 29	Australia, 2016	Primary data collection and analysis: Multivariable logistic regression to assess correlates of risky drinking	MUP/pricing policy	**Introducing/increasing MUP decreased alcohol consumption in older adults**. Respondents who were older, in a higher SES group, or unemployed had increased odds of risky drinking. Approximately 54% of people reporting risky drinking experienced a negative outcome as a result of their drinking in the last year.
Leal-López et al. 2020 36	Europe and North America, 2001–2002, 2005–2006, 2009–2010, and 2013–2014	Secondary data collection and analysis: Multilevel modeling of four waves of repeat cross-sectional survey data	National alcohol control policies	**National alcohol control policies mitigated harmful alcohol behaviors**. Combinations of stronger alcohol policies that reduced alcohol affordability also reduced alcohol-related problems among adolescents from a range of socioeconomic backgrounds, thereby reducing inequality in alcohol outcomes.
Myran et al. 2019 31	Canada, 2013–2014, 2016–2017	Secondary data collection and analysis: Health administrative records	Alcohol outlet density/clusters, on-/off-premise policies	**Increased hours of operation of alcohol outlets and numbers of alcohol outlets were associated with worsened health**. Increased hours of operation and numbers of alcohol outlets within defined geographic regions (forward sortation areas [FSAs]) were positively associated with higher rates of alcohol-attributable emergency department (ED) visits. The increase in ED visits attributable to alcohol was 6% greater in FSAs that introduced alcohol sales in grocery stores following deregulation compared with FSAs that did not.
**Studies including racial/ethnic subgroups**				
Clough et al. 2016 24	Australia, 2013–2015	Primary data collection and analysis: Interviews	National alcohol control policies	**Participants attributed reduced violence and improved community amenity to alcohol management plans (complex alcohol control strategies)**.
Hadland et al. 2015 15	United States, Alcohol Policy Scale 1999–2008, and age-adjusted alcoholic cirrhosis death rates 3 years later	Secondary data collection and analysis: Special focus on American Indian/Alaska Native (AI/AN) groups	Taxation, access/availability reduction policies	**Stronger alcohol policies generally were associated with lower alcoholic cirrhosis death rates among females**. This was not found among AI/AN populations, who experienced higher rates in the aggregate. Tribal alcohol control policies often differ from state policies, which may not reach rural and reservation lands to the same extent.
Roberts et al. 2018 18	United States, 1985–2016	Secondary data collection and analysis: Drinking and demographic data from pregnant women	Priority treatment for pregnant women and women with children, prohibiting prosecution of pregnant women testing positive for substance use, mandatory warning signs, and other relevant policies, as listed by states in the Alcohol Policy Information System	**A variety of policies aimed at reducing drinking during pregnancy affected women from different racial/ethnic groups differently**. Priority treatment for pregnant women and women with children was associated with less heavy drinking by pregnant Black women; prohibitions on criminal prosecution of pregnant women testing positive for alcohol use were associated with less heavy drinking by pregnant White women.
Roberts et al. 2019 17	United States, 1972–2015	Secondary data collection and analysis: Data from more than 150 million singleton births in Vital Statistics.	Priority treatment for pregnant women and women with children; prohibiting prosecution of pregnant women testing positive for substance use; mandatory warning signs; and other relevant policies as listed by states in the Alcohol Policy Information System Outcomes: Birth outcomes (e.g., preterm births, low birthweight and prenatal care utilization)	**Policies aimed at reducing drinking during pregnancy affected birth outcomes for Black and White women differently**. Alcohol/pregnancy policies had adverse effects for White women (i.e., increased rates of preterm births and/or low birth weight), whereas they were more beneficial for reducing preterm births in Black women.
Silver et al. 2019 20	United States, 2004–2010	Secondary data collection and analysis	State alcohol policy scores Outcomes: Drinking patterns (e.g., binge or 10+ drinks/occasion) by race/ethnicity	**All population groups showed declines in binge drinking with increasing alcohol policy scores**. There were no statistically significant differences between population groups.
Subbaraman et al. 2021 21	United States, 1999–2016	Secondary data collection and analysis	Beverage-specific taxes and government control of spirits retail Outcomes: Alcohol-attributable mortality rates	**Beverage-specific taxes were not associated with an increase in alcohol-attributable mortality rates**. However, government control of spirits retail was associated with reductions in alcohol-associated mortality rates (3% reductions in Whites and overall, versus 4% reductions in Hispanics). Caveat: Alcohol taxes were relatively low; thus, it was hard to see much of an effect.
**Studies including both SES-based and racial/ethnic subgroups**				
Balasubramani et al. 2021 30	India, 2015–2016	Secondary data collection and analysis	National alcohol control policies	**National alcohol control policies mitigated harmful alcohol behaviors**. Socioeconomic factors had a low to moderate effect on alcohol consumption; most districts with high consumption of alcohol, especially by women in alcohol hot spots, coincided with districts with a high proportion of scheduled Tribe population; a strong association existed between women, scheduled Tribe population, and alcohol consumption; high alcohol consumption was also seen among women in the poorest groups, individuals with no education, and manual/agricultural workers.
Conigrave et al. 2020 25	Australia, 1988–2018	Secondary data collection and analysis: Systematic literature review	National alcohol control policies	**National alcohol control policies mitigated harmful alcohol behaviors**. Indigenous Australian drinking patterns varied within and between communities. Initiatives/alcohol policies to reduce high-risk drinking need to account for this variability.
Greenfield et al. 2019 14	United States, alcohol surveys in 2000, 2005, 2010, and 2015	Primary and secondary data collection and analysis	Expert panel assessments of state alcohol policy scale Outcomes: Longitudinal alcohol use and 2015 reports of collateral damage from alcohol (alcohol-related harm to others)	**National alcohol control policies mitigated harmful alcohol behaviors**. For people under age 40, in states with lower scores on the Alcohol Policy Scale (i.e., policies were judged to be less effective in reducing binge drinking and impaired driving) those with SES below federal poverty level and American Indians experienced more alcohol-related harms to others, whereas those with a college degree or higher were much less likely to experience alcohol-related harms to others.
Naimi et al. 2016 16	United States, 2011	Secondary data collection and analysis: State-level survey data	Taxation	**State-specific alcohol tax increases would cost more for excessive drinkers, those with higher incomes, and Whites**. Members of low-income households and racial/ethnic minorities, including those who did not drink excessively, paid less in per capita and aggregate costs following tax increases, compared with non-Hispanic Whites or those with higher incomes. Increasing the price of alcohol might be a particularly effective way to reduce the risk of alcohol-related harms in low-income populations.
Shoesmith et al. 2016^32^	Malaysia, prior to January 2016	Primary data collection and analysis: Mixed methods	Taxation	**Low taxes were linked to more alcohol consumption**. Inexpensive, untaxed alcohol (called Montoku) was consumed by people with very heavy episodic drinking, which argued for an excise tax in the form of a specific rate per liter of alcohol.
Silver et al. 2022 19	United States, 2011–2019	Secondary data collection and analysis	State alcohol policy scores	**Alcohol control policies mitigated harmful alcohol behaviors**. States with stronger alcohol policies had comparatively lower heavy drinking rates, but only for those with less than high school education, and particularly for Whites. As alcohol policy scores increased, the heavy drinking disparities between racial/ethnic groups and education groups diminished.
Subbaraman et al. 2020 6	United States, 2000–2015	Primary and secondary data collection and analysis	State-level alcohol taxation, density of on- and off-premise outlets, and demographics of participants Outcomes: Alcoholic beverage volume and alcohol-related consequences	**An increase in taxes was associated with a decrease in alcohol consumption**. Higher beer tax led to lower alcohol volume and fewer alcohol-related consequences for Black women in particular. Higher spirits taxes reduced alcohol volume among Hispanic women and men. Alcohol availability policies resulting in lower bar densities affected White men most. Overall, both beverage-specific taxes and policies influencing on- and off-premise sales appeared to affect different subgroups variably.
Trangenstein et al. 2020 22	United States, 2016	Secondary data collection and analysis	Alcohol outlet density/clusters, on-/off-premise policies	**An increase in alcohol outlet clusters was associated with worsened health**. The most robust predictor of alcohol outlet cluster membership was a history of redlining (i.e., racially discriminatory lending policies). Level of economic investment (marked by vacant properties) appeared to be a key characteristic that separated census block groups (CBGs) in on- and off-premise outlet clusters. CBGs with racial/ethnic or socioeconomic advantage had higher odds of being in on-premise clusters, and CBGs marked by disinvestment had higher odds of being in off-premise clusters.

Articles are grouped by population type, and then by author names in alphabetical order.
